# Case Report: A Rare Case of a Combination of Omphalocele With Umbilical Teratoma

**DOI:** 10.3389/fped.2021.726593

**Published:** 2021-09-13

**Authors:** Ruslan Bilal, Dastan Rustemov, Zhenis Sakuov, Bahytkaly Ibraimov, Arman Kozhakhmetov

**Affiliations:** ^1^Department of Medicine, School of Medicine, Nazarbayev University, Nur-Sultan, Kazakhstan; ^2^Department of Pediatric Surgery, National Research Mother and Child Center, Nur-Sultan, Kazakhstan

**Keywords:** omphalocele, teratoma, newborns, malformation, congenital

## Abstract

Omphalocele is a congenital malformation of the abdominal wall, which occurs with a frequency of 1–5,000 newborns. The prognosis of treatment often depends on the presence of concomitant malformations. The most common contents of the hernia with omphalocele are the intestinal loops, liver, spleen. However, all organs of the abdominal cavity can be part of the hernial sac with large sizes of omphalocele. Teratoma is a germ cell tumor made up of several different types of tissue, such as hair, muscle, teeth, or bone. They are a type of germ cell tumor and divided into two types: mature and immature. In this article, we describe a rare case of a combination of an omphalocele with a mature teratoma and report the successful single step surgical treatment. On the first day after birth, a simultaneous operation—Removal of teratoma with abdominoplasty was performed. The postoperative period was uneventful, and the child was discharged for recovery.

## Introduction

Omphalocele is a congenital malformation of the anterior abdominal wall, which has a central localization and is characterized by the presence of a hernial sac with contents inside. Most often, the contents of the hernial sac are the liver, and loops of the small or large intestine. The incidence of omphalocele varies from 1 in 4,000 to 1 in 10,000 (1–3). In comparison with other malformations of the anterior abdominal wall, omphalocele is often combined with other congenital malformations of the cardiovascular, respiratory or gastrointestinal tracts. Approximately 40% of patients with omphalocele have an abnormal karyotype (4).

Teratoma is a germ cell tumor, which is most often localized in the sacrococcygeal region (89%). Teratomas can be cystic, cystic-solid, or mixed. They can also be found in other areas. Congenital teratomas are most common benign formations, although in recent years there have been reports of early malignancy of the formation.

Surgical treatment for small and medium-sized omphalocele without viscero-abdominal imbalance consists of a one-stage reconstruction of the anterior abdominal wall. With a large defect and an insufficient volume of the abdominal cavity, treatment consists in a two-stage immersion of the organs as the volume of the abdominal cavity increases.

We describe a clinical case of successful single step surgical treatment of omphalocele in combination with umbilical cord teratoma.

## Case Presentation

**Figure 1:**
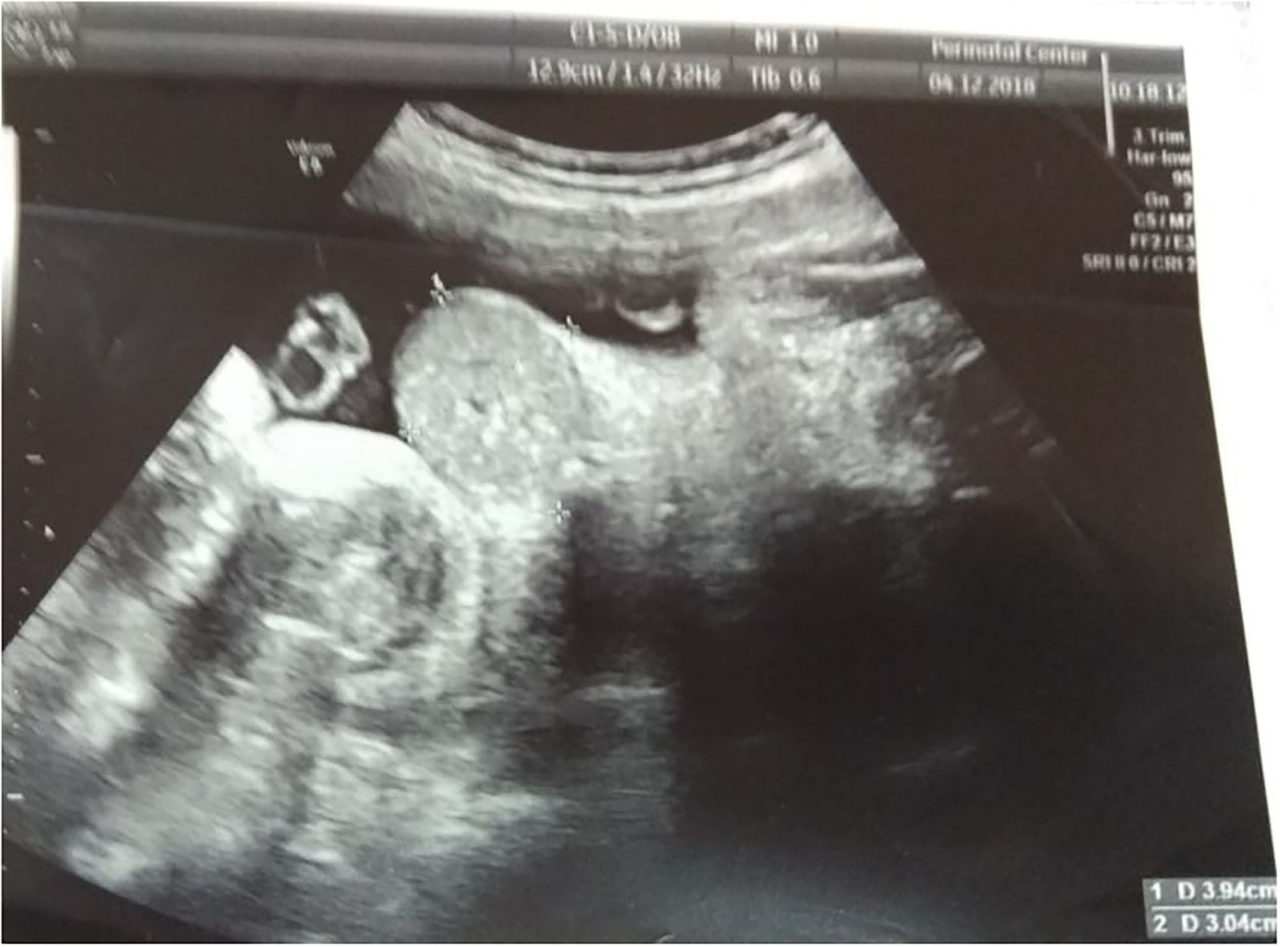
Prenatal US on 34 weeks of gestation.

**Figure 2:**
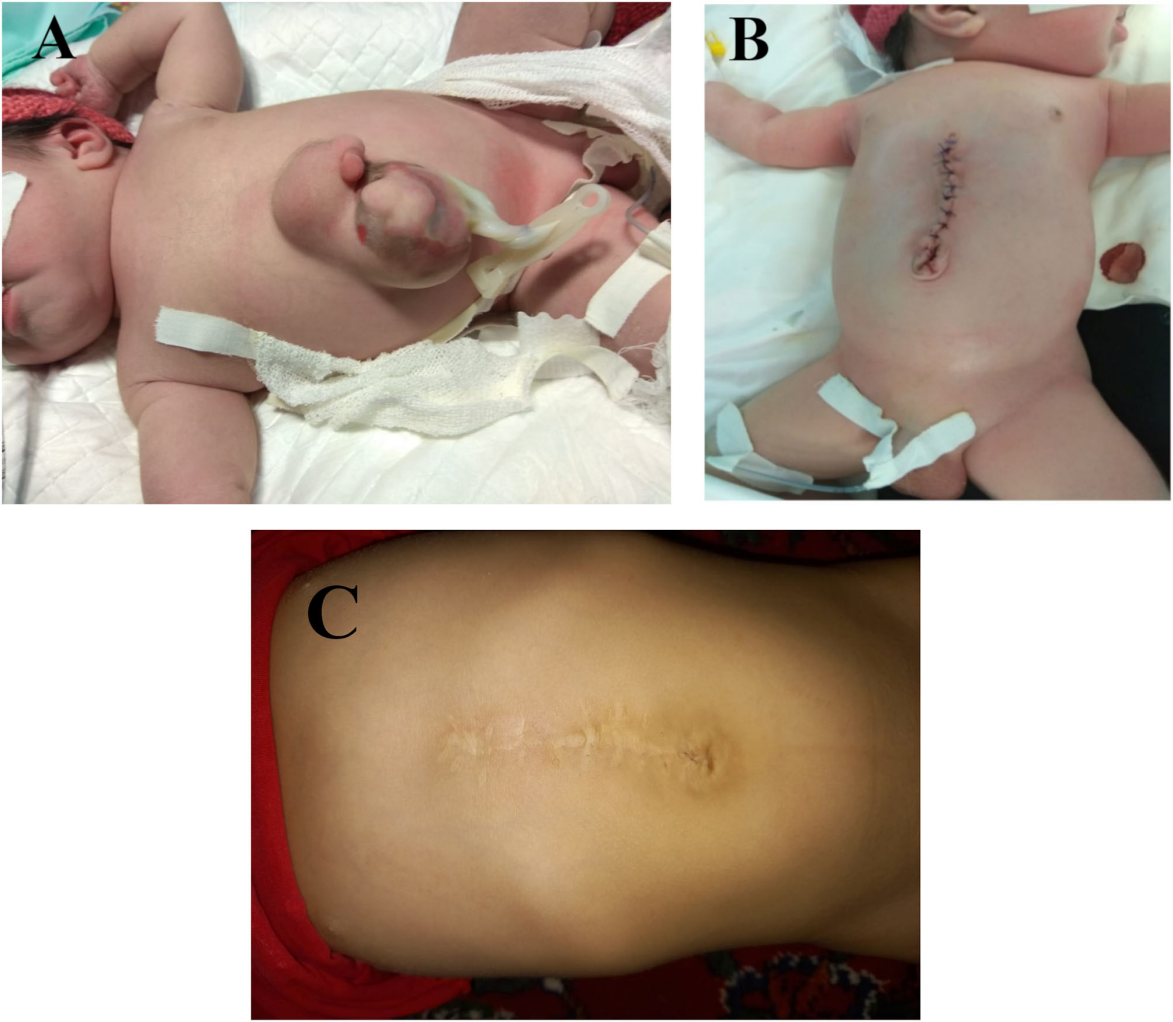
**(A)** A defect of the anterior abdominal wall in the umbilical area measuring 4.0 × 5.0 cm, covered with a membrane and skin along the periphery. In the center of the membrane there was an area of unchanged skin with the size of 2.0 × 2.0 cm. **(B)** The surgical wound on the 3rd postoperative day. **(C)** The postoperative scar on the recent follow up.

The diagnosis of omphalocele was made prenatally based on ultrasound findings. The woman was referred to the Center of motherhood and childhood for childbirth. The mother of the baby had had total thyroidectomy 3 years before the start of pregnancy with postoperative hypothyroidism, and had moderate anemia and impaired uteroplacental blood flow (1A degree). Antenatally, at 25 weeks the fetus was diagnosed with a congenital malformation of the anterior abdominal wall. At 34 weeks, the diagnosis of a small omphalocele was confirmed by ultrasound. No other abnormalities of the abdominal wall were detected (Figure 1). Urgent operative delivery by ceserean section at 39 weeks of gestation was performed. A male child was born, birth weight-−3,600 g, height-−54 cm. Assessment on the Apgar scale was 8/9 points. At birth, in the umbilical area, a defect of the anterior abdominal wall measuring 4.0 × 5.0 cm, covered with a membrane and skin along the periphery, was found. In the center of the membrane there was an area of unchanged skin with the size of 2.0 × 2.0 cm (Figure 2A). Karyotype analysis results-−46, XY. The omphalocele contained intestinal loops. Above the omphalocele, surgical team detected a tumor-like formation of 3.0 × 3.0 cm with soft elastic consistency and skin growths covered with villus hair. On ultrasound, intestinal loops were in the area of the hernial sac.

Surgical treatment was performed 15 h after birth following stabilization of the newborn's condition and correction of water-electrolyte balance and impaired coagulation. The omphalocele was surgically removed with tumor-like formation on the external surface of the omphalocele 3.0 × 3.0 cm in size with even margins and no invasion into adjacent tissues. Inside, a structure of bony-cartilaginous consistency was palpated. Since there was a sufficient volume of the abdominal cavity, one-stage plastic surgery of the anterior abdominal wall was performed. The postoperative period was uneventful. On the third post-operative day there were no signs of inflammation and dehiscence of the surgical wound (Figure 2B) and it eventually healed by primary intention. The child was discharged on the 11th day after surgery. On the most recent follow-up examination the baby is in a good state, developing according to his age. There are no complications of postoperative scar (Figure 2C).

**Figure 3:**
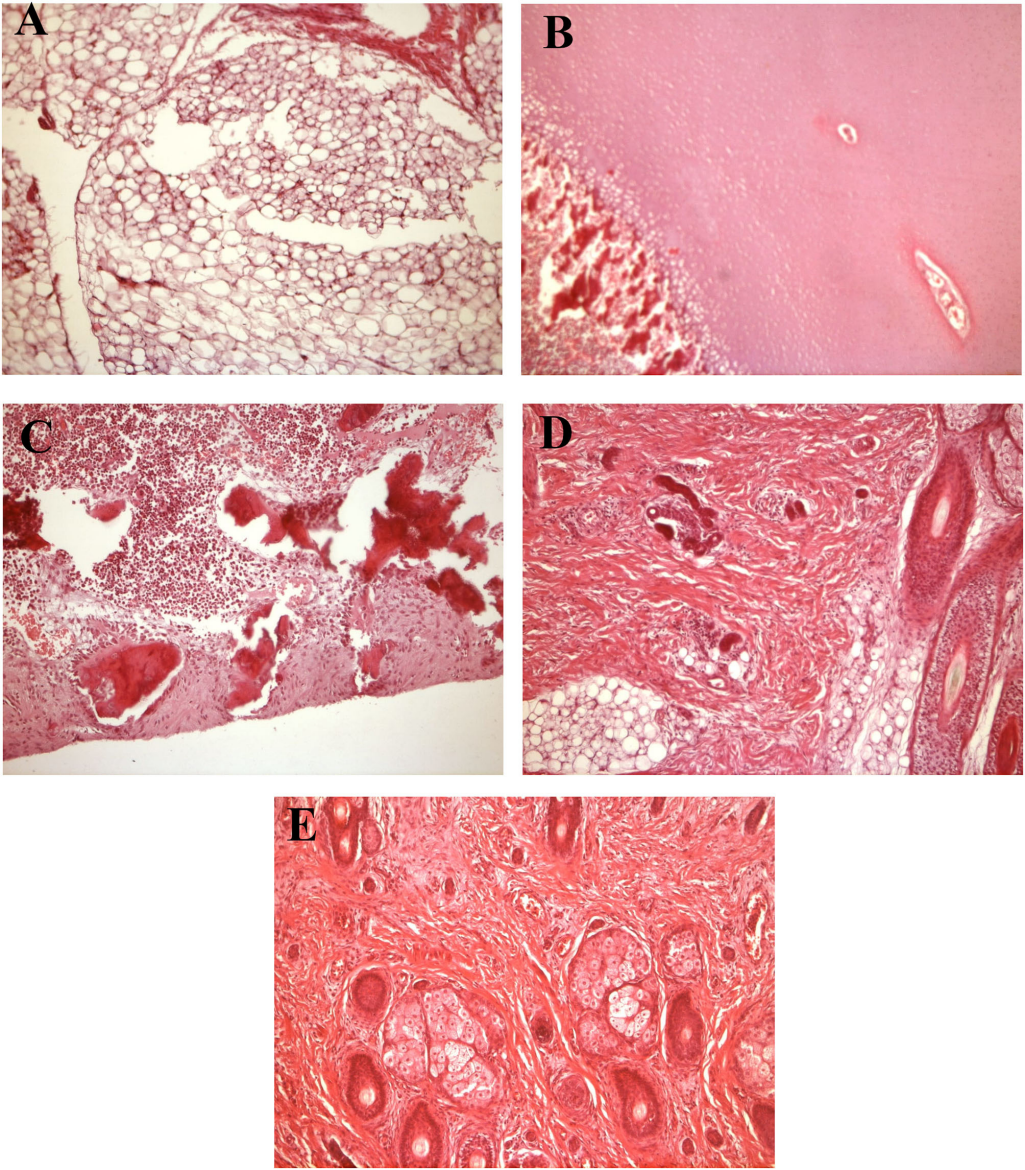
Histology results. **(A)** Adipose tissue. **(B)** Cartilaginous tissue. **(C)** Bone marrow. **(D)** Hair follicles. **(E)** Single sebaceous glands.

Histology revealed islets of adipose (Figure 3A), cartilaginous (Figure 3B), bone tissue, bone marrow (Figure 3C), hair follicles (Figure 3D) and single sebaceous glands (Figure 3E)—a mature teratoma.

## Discussion

The medical literature describes rare cases of congenital umbilical cord teratomas (5). In our clinical case, the child had a combination of a mature teratoma with a congenital malformation of the anterior abdominal wall—omphalocele. We share our experience of successful surgical treatment of a newborn with this extremely rare combination.

One-third of newborns with omphalocele detected in the antenatal period have related developmental abnormalities (1). The frequency of malformation ranges from 1:5,000-1:10,000 live-born children (6). Congenital teratomas of the umbilical cord are extremely rare and only 15 cases have been reported in the literature. Five cases included a combination of umbilical cord teratoma and omphalocele (7), with two early abortions due to chromosomal abnormalities.

In our Center, over the past 5 years three newborns with malignant forms of sacrococcygeal teratomas have been observed. Histologically, teratomas are often constituted of embryonic cells of all three germ layers.

In this case, omphalocele and teratoma despite being two different pathologies were managed as one entity at the same time. Since there was no significant viscero-abdominal disproportion, surgical treatment was performed in one stage by removal of the teratoma with simultaneous closure of the abdominal cavity.

Congenital omphalocele needs to be differentiated from gastroschisis, which is also a congenital malformation of the anterior abdominal wall. Unlike omphalocele, the defect in gastroschisis is most often located to the right of the normally formed umbilical cord, and the hernial sac is absent in most cases. Despite the pronounced changes in the internal organs in gastroschisis and the presence of viscero-abdominal imbalance, the prognosis following treatment is most often favorable. The survival rate in this group of patients in our Center in 2020 was 97%.

In conclusion, combination of the omphalocele and the teratoma that we have described is extremely rare and difficult to diagnose antenatally. This malformation is correctable and does not constitute an indication for the termination of pregnancy.

## Data Availability Statement

The original contributions presented in the study are included in the article/supplementary material, further inquiries can be directed to the corresponding author/s.

## Ethics Statement

Written informed consent was obtained from the minor(s)' legal guardian/next of kin for the publication of any potentially identifiable images or data included in this article.

## Author Contributions

RB consulted in the prenatal period, participated in the post-operative period, designed, and wrote the draft. DR and ZS performed surgery and provided post-operative care. BI participated in the data collection and provided histology analysis. AK reviewed the literature and participated in the publication. All authors contributed to the article and approved the submitted version.

## Funding

This study received financial support from Nazarbayev University School of Medicine (NUSOM).

## Conflict of Interest

The authors declare that the research was conducted in the absence of any commercial or financial relationships that could be construed as a potential conflict of interest.

## Publisher's Note

All claims expressed in this article are solely those of the authors and do not necessarily represent those of their affiliated organizations, or those of the publisher, the editors and the reviewers. Any product that may be evaluated in this article, or claim that may be made by its manufacturer, is not guaranteed or endorsed by the publisher.
